# Deep neural networks and genome-wide associations reveal the polygenic architecture of local brain aging

**DOI:** 10.1007/s11357-025-02046-1

**Published:** 2025-12-11

**Authors:** Nicholas J. Kim, Ayati Mishra, Nahian F. Chowdhury, Samuel D. Anderson, Owen M. Vega, Nikhil N. Chaudhari, Kenneth H. Buetow, Paul M. Thompson, Andrei Irimia

**Affiliations:** 1 Alfred E. Mann Department of Biomedical Engineering, Viterbi School of Engineering, University of Southern California, Los Angeles, CA USA; 2 Ethel Percy Andrus Gerontology Center, Leonard Davis School of Gerontology, University of Southern California, Los Angeles, CA USA; 3 School of Life Sciences Center for Social Dynamics and Complexity, Arizona State University, Tempe, AZ USA; 4 Imaging Genetics Center, Mark and Mary Stevens Neuroimaging and Informatics Institute, University of Southern California, Marina del Rey, CA USA; 5 Department of Quantitative & Computational Biology, Dornsife College of Arts and Sciences, University of Southern California, Los Angeles, CA USA; 6 Centre for Healthy Brain Aging, Institute of Psychiatry, Psychology & Neuroscience, Department of Psychological Medicine, King’s College, London, England UK

**Keywords:** Brain age, Deep learning, MRI, GWAS

## Abstract

Local brain age (LBA) is a regional metric of brain aging that offers a spatially resolved alternative to global brain age, but whose genetic basis is unexplored. This study reports the first genome-wide association study of cortical LBA, as estimated by a deep neural network from the *T*_*1*_-weighted magnetic resonance images of 41,708 cognitively normal adults in the UK Biobank. We identified 1,212 single-nucleotide polymorphisms (SNPs) significantly associated with LBA in at least one brain region. Genes mapped to these SNPs are involved in developmental, metabolic, immune, and cytoskeletal pathways. Dimensionality reduction of SNP association profiles uncovered three clusters linked to morphogenetic, cytoskeletal, and immuno-epigenetic processes, helping to relate neuroanatomic, immunosenescent and epigenetic mechanisms of brain aging. Top variants are mapped to *KCNK2*, *NUAK1*, *GMNC*, *MSL2*, and to other genes implicated in neurodevelopment and neurodegeneration. Spatial clustering of LBA-associated variants in default mode, limbic, and motor network regions parallel regional vulnerability to Alzheimer’s disease and frontotemporal dementia. These findings establish a polygenic architecture for regional brain aging and support LBA as a genetically informed phenotype for studying aging-related neurodegeneration. Our results suggest that cortical aging is not governed by isolated loci but by coordinated genetic programs—rooted in development, metabolism, and cellular structure—that confer lifelong patterns of regional brain vulnerability and resilience. This first genetic dissection of spatially specific brain aging reveals a polygenic landscape of coordinated genetic programs, developmentally encoded and metabolically maintained during senescence. This study reframes aging as an anatomically specific genetic process reflecting the varying structural vulnerabilities observed across neurodegenerative diseases.

**Supplementary Information:**

The online version contains supplementary material available at 10.1007/s11357-025-02046-1.

Global brain age (GBA) quantifies the structural age of the brain, which may differ from an individual's chronological age (CA) [1]. GBA is typically inferred using machine or deep learning models trained on magnetic resonance images (MRIs). Deviations of GBA from CA quantify whether brain aging is slower or faster than expected, the latter indicating higher neurodegenerative risk [2]. GBA gap (GBAG), defined as the difference between estimated GBA and CA, has been used to identify individuals at elevated risk for cognitive decline, Alzheimer's disease (AD), Parkinson’s disease (PD), amyotrophic lateral sclerosis (ALS), and other neurodegenerative conditions [1, 2]. However, GBAG provides only one summary measure and fails to account for, or to quantify, the well-documented regional variation of brain aging [3]. To address this limitation, local brain age (LBA) quantifies BA at each MRI voxel or anatomically defined region [3, 4]. LBA offers a spatially resolved phenotype of neural aging and a more nuanced analysis of region-specific senescence.

The genetic architecture of LBA has not been studied. Genome-wide association studies (GWASs) have identified genetic variants (single-nucleotide polymorphisms, SNPs) associated with GBA phenotypes [5], but no study has extended this approach to spatially localized measures such as LBA. Understanding the genetic basis of LBA could provide novel insights into the biological mechanisms that govern region-specific brain aging and its relation to neurodegenerative disease. In this study, we conducted the first GWAS of LBA in 41,708 cognitively normal individuals in the UK Biobank (UKBB). Specifically, we investigated LBA gap (LBAG), which captures the deviation of LBA from CA at each MRI voxel or region, using a deep neural network (DNN) to estimate voxel-wise LBA from *T*_*1*_-weighted MRIs. LBA estimates were mapped to 148 brain regions, and CA was subtracted to compute region-wise LBAGs. We then tested associations between LBAGs and 662,971 SNPs, identifying 1,212 genome-wide significant variants linked to at least one brain region. Gene mapping and dimensionality reduction analyses uncovered biologically coherent SNP clusters involved in developmental, cytoskeletal, metabolic, and immune-epigenetic processes. Our findings establish a polygenic architecture for regional brain aging and provide a framework for understanding how genetic variation may contribute to the spatial heterogeneity of cortical aging. By moving GWASs of BA from a single number (GBA) to a high-resolution brain map (LBA), this research suggests a new program to synergize personalized genetics, geoscience, and neurology.

## Results

### GWAS

**Fig. 1:**
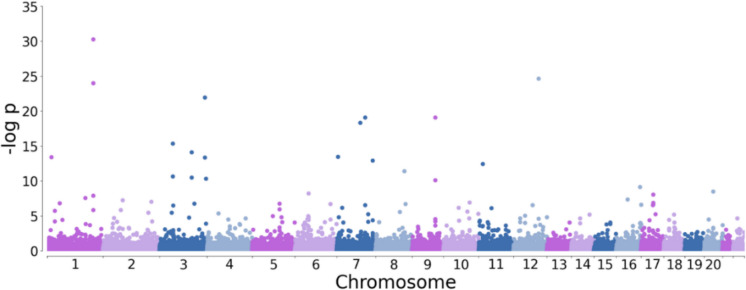
Manhattan plot for associations between SNPs and LBAG

**Fig. 2:**
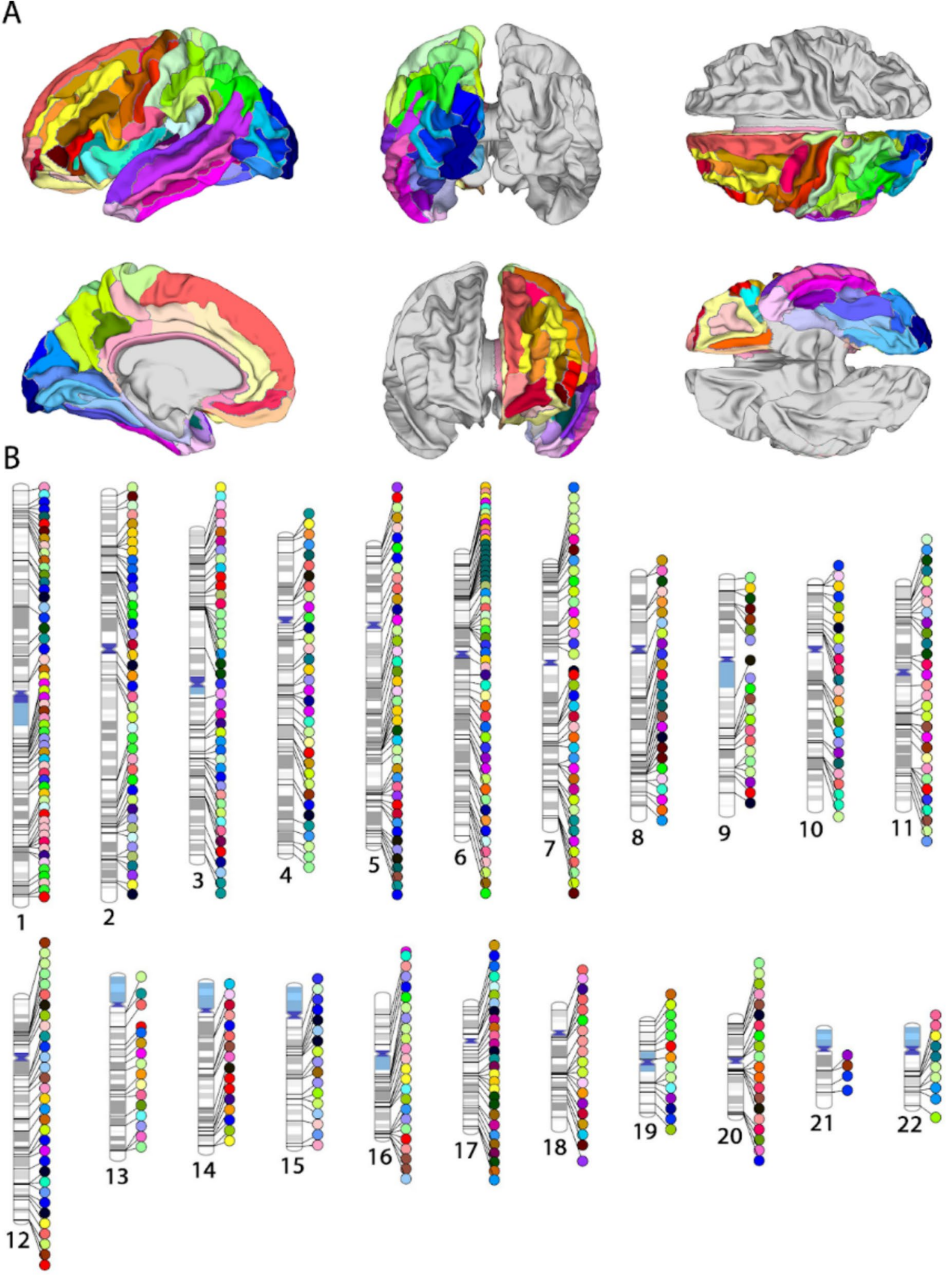
Genetic associations with LBAG for the left hemisphere. (**A**) Cortical map showing the left cerebral cortex color-coded by distinct anatomical regions. Colors indicate the cortical region where each SNP exhibits its strongest association with LBAG. (**B**) Phenogram mapping these SNPs to their chromosomal locations. Colored dots represent individual SNPs, with colors corresponding to the cortical regions from (A) where their association with LBAG is strongest. The color key for cortical regions is shown in Fig. 4

**Fig. 3:**
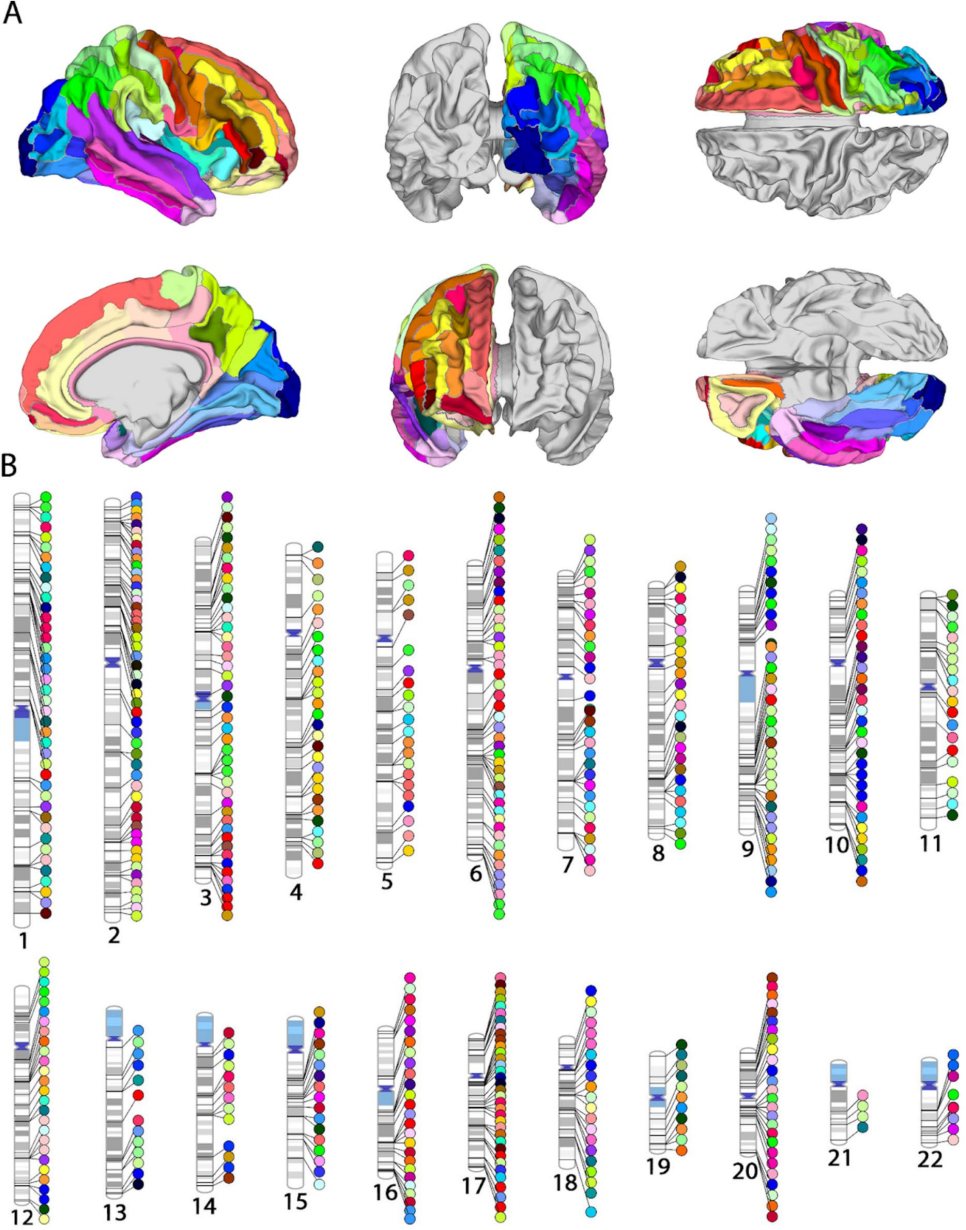
Genetic associations with LBAG for the left hemisphere. (**A**) Cortical map showing the right cerebral cortex color-coded by distinct anatomical regions. Colors indicate the cortical region where each SNP exhibits its strongest association with LBAG. (**B**) Phenogram mapping these SNPs to their chromosomal locations. Colored dots represent individual SNPs, with colors corresponding to the cortical regions from (A) where their association with LBAG is strongest. The color key for cortical regions is shown in Fig. 4

**Fig. 4:**
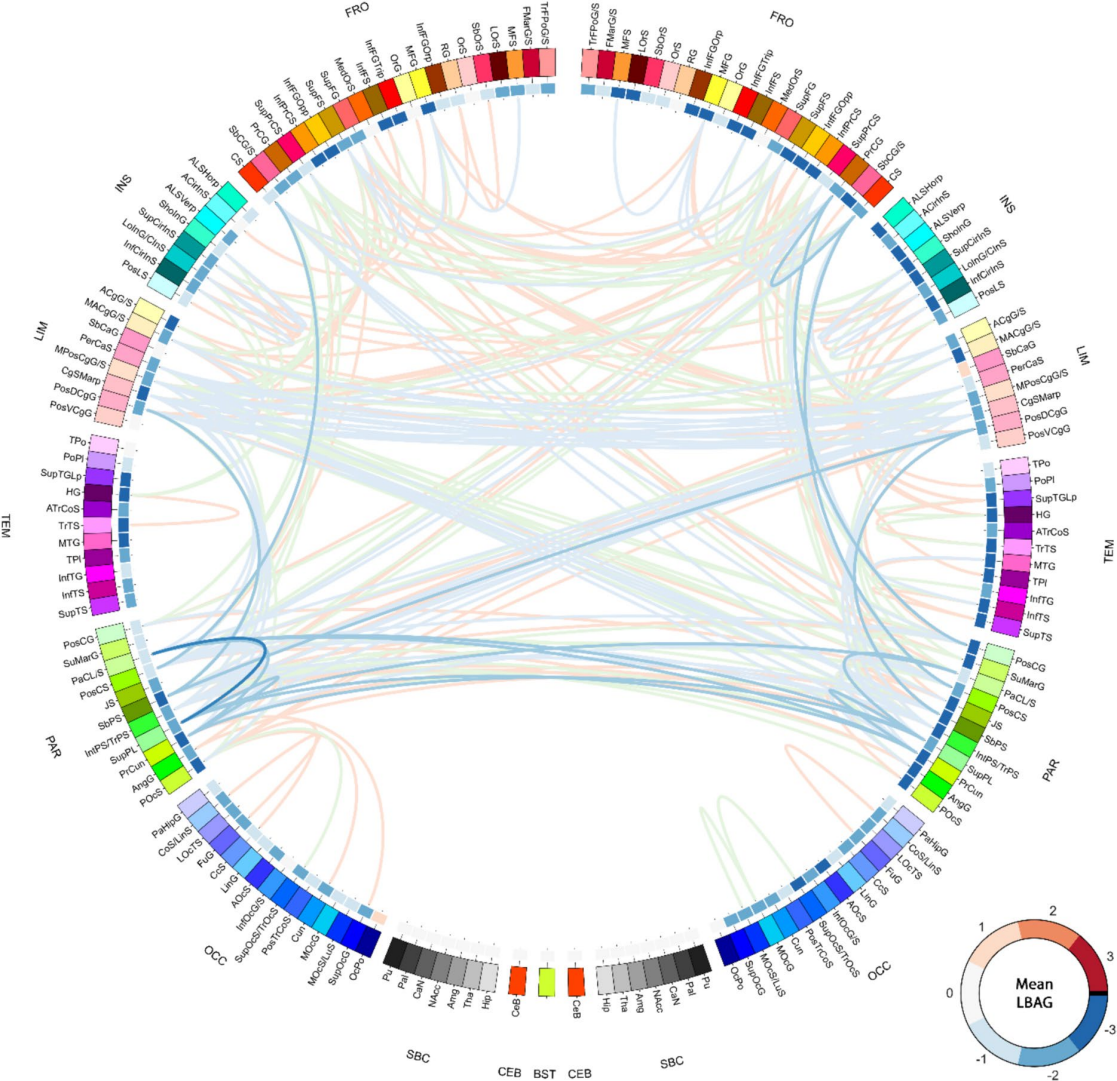
Connectogram of shared genetic associations with LBAG. The outer ring represents 148 anatomically defined cortical regions, grouped by lobe (FRO: frontal, INS: insular, LIM: limbic, TEM: temporal, PAR: parietal, OCC: occipital, SBC: subcortical, CEB: cerebellar). The left half of the connectogram corresponds to the left hemisphere, and the right half to the right hemisphere. The inner heatmap ring represents the mean LBAG across all subjects for each cortical region, with blue indicating younger-appearing regions, red indicating older-appearing regions, and white representing age-typical appearance. To enhance interpretability, only connections with ≥ 30 shared SNPs were included. Connections are color-coded by strength: red indicates 30–34 shared SNPs, green indicates 35–40, and blue indicates more than 40 shared SNPs, with darker hues denoting stronger genetic overlap

Our genetic analysis identifies 1,212 SNPs exhibiting significant genome-wide associations with LBAG in at least one cortical region after adjusting for linkage disequilibrium (LD) and for the false discovery rate (FDR, Supplementary Data 1). The mean genomic inflation factor $\overline{\uplambda }$ across all cortical regions, calculated prior to FDR correction, is 0.92 (range: 0.87–0.97), indicating slight deflation of test statistics. Supplementary Fig. 1 presents a histogram of λ values across cortical regions. All subsequent analyses focus on the set of clumped, FDR-corrected SNPs. A Manhattan plot highlights the statistical significance of each SNP’s most significant *β* regression coefficient for association with LBAG (Fig. 1). The most strongly associated SNPs are located on chromosomes 1, 3, 7, 9, and 12. Phenograms map LBAG-associated SNPs to their chromosomal locations alongside cortical regions where their associations are strongest. Figure 2A displays the left hemisphere (LH)’s cortical map, while Fig. 2B presents the corresponding SNP locations across chromosomes. Similarly, Fig. 3A depicts the right hemisphere (RH)’s cortical map, with Fig. 3B indicating associated chromosomal positions. Genetic associations with LBAG are distributed across all autosomes and encompass all cortical regions. In the LH, the paracentral lobule and sulcus exhibit the highest number of significant SNP associations with LBAG, while the lateral orbital sulcus has the fewest. In the RH, the paracentral lobule and sulcus also exhibit the greatest number of associations, and the occipital pole the fewest. Pairwise connections between cortical regions that share at least 30 significantly associated SNPs are visualized using a connectogram (Fig. 4). All regions exhibit positive LBAGs except for the left occipital pole and right subcallosal area. Strong inter-regional genetic overlap is observed for LBAG-associated SNPs. The most prominent of these relationships are observed between bilateral homologous regions within the frontal, parietal, and limbic lobes. Dense relationships among regions such as the superior frontal gyrus, precentral sulcus, paracentral lobule, and cingulate cortex indicates a symmetric and midline-dominant genetic influence on brain aging. Additionally, strong fronto-parietal and fronto-limbic links—particularly between medial frontal areas, the precuneus, and posterior cingulate—indicate a shared polygenic architecture along the dorsal and midline cortical surface.

### *KCNK2* and *NUAK1* have opposing effects on the polygenic architecture of cortical aging

**Fig. 5:**
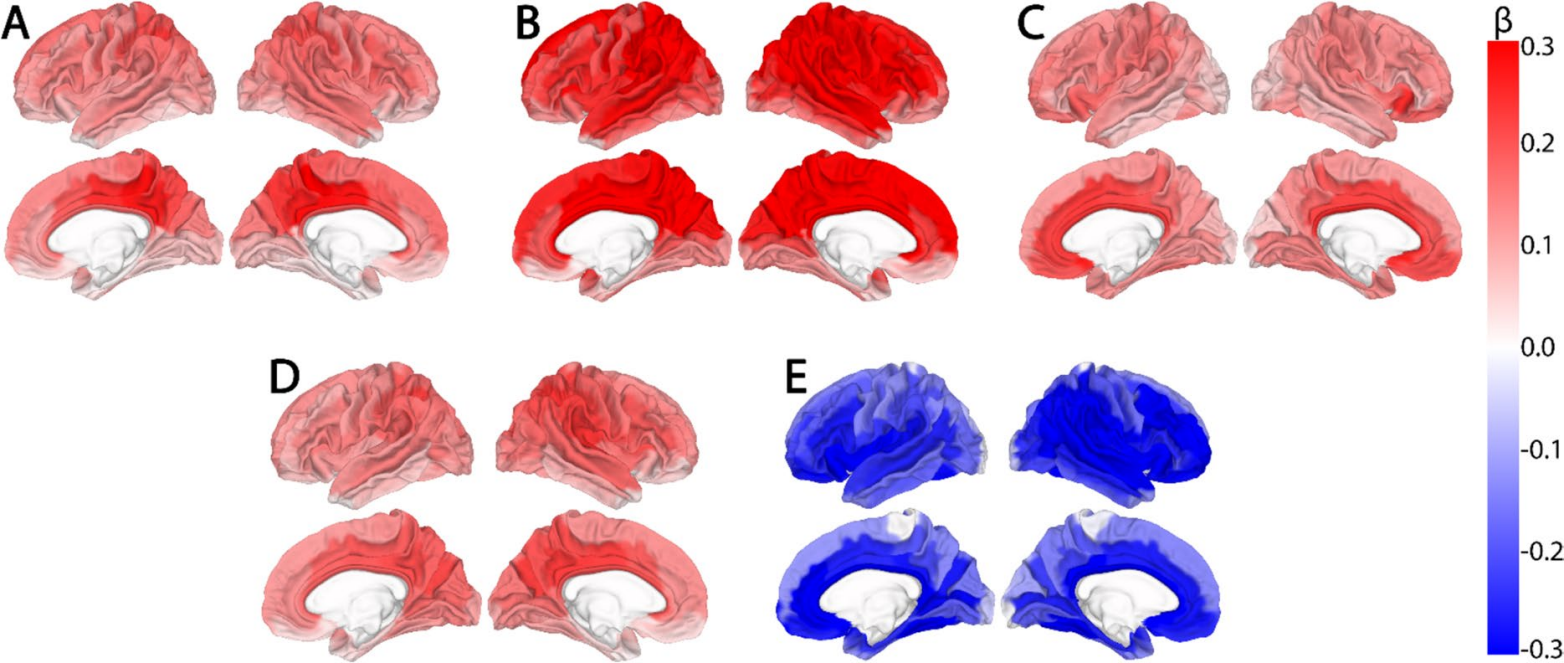
Cortical maps of β coefficients for five top-ranking SNPs associated with LBAG. Red: positive associations (higher LBAG with more reference alleles); blue: anticorrelation. This set of five SNPs includes (**A**) rs864736, KCNK2; (**B**) rs59084003, KCNK2; (**C**) rs13089287, GMNC; (**D**) rs1393786, MSL2; and (**E**) rs12146713, NUAK1. All are found among both the top 10 highest-impact and top 10 broadest-impact variants in our study

Among the variants most strongly associated with LBAG, five (rs864736, rs59084003, rs13089287, rs1393786, and rs12146713) rank within both the top 10 highest-impact (Table 1) and broadest-impact (Table 2) variants. Two of these SNPs, rs864736 and rs59084003, map to the potassium two-pore domain channel subfamily *K* member 2 (*KCNK2*) gene but are not in LD and represent independent association signals. rs864736 lies 29 kilobases (kb) downstream of *KCNK2*, and rs59084003 lies 25 kb downstream. These SNPs have broadly similar patterns of association with LBAG (Fig. 5A for rs864736; Fig. 5B for rs59084003). Both exhibit significant widespread bilateral associations concentrated in the cingulate and parietal cortices, most notably in the marginal branches of the cingulate sulci, in posterior cingulate cortices, subparietal sulci, precunei, and intraparietal sulci. All associations involve positive *β* coefficients, indicating that each additional copy of the reference allele is associated with higher LBAG and with an older-appearing cortex. Notably, rs59084003 has stronger associations with LBAG in sensorimotor regions such as the central and postcentral sulci, while rs864736 exhibits the most significant *β* coefficient for association with LBAG across all examined variants. Together, these findings suggest that *KCNK2* variants exert strong, widespread influences on cortical aging, promoting older-appearing morphology.

**Table 1:** Ten highest-impact SNPs according to their most significant *β* coefficient (-log *p* > 13.5) for association with LBAG

Gene(s)	SNP	Chr	Position	Allele	MAF	β	-log p	Coverage (%)	Function
KCNK2	rs864736	1	215,150,260	C/A	0.45	0.310	30.3	68.00	Two-pore-domain background potassium channel
	rs59084003	1	215,154,276	C/T	0.93	0.534	24.0	88.67	
NUAK1	rs12146713	12	106,476,805	T/C	0.91	−0.397	24.6	70.67	Serine/threonine-protein kinase
GMNC	rs13089287	3	190,654,424	T/C	0.62	0.270	22.0	78.00	Multiciliate cell development
WNT16	rs2707466	7	120,979,089	C/T	0.55	0.198	19.1	42.67	Cell patterning in embryogenesis
LPAR1	rs1409682	9	113,665,419	G/A	0.79	−0.252	19.1	19.33	Lysophosphatidic acid receptor
SLC25A13	rs1880512	7	96,215,832	C/T	0.32	−0.264	18.3	27.33	Aspartate/glutamate antiporter
SLC6A20	rs17279437	3	45,814,094	G/A	0.90	0.317	15.4	31.33	Neurotransmitter symporter
MSL2	rs1393786	3	135,854,035	C/A	0.74	0.236	14.1	70.67	DNA damage response
GNA12	rs798489	7	2,801,803	C/T	0.73	0.201	13.5	17.33	Protein phosphatase activator

**Table 2:** Ten broadest-reaching SNPs according to their number of cortical locations significantly associated with LBAG

Gene(s)	SNP	Chr	Position	Allele	MAF	β	-log p	Coverage (%)	Function
NUAK1	rs12146713	12	106,476,805	T/C	0.91	−0.397	24.6	88.67	Serine/threonine-protein kinase
GMNC	rs13089287	3	190,654,424	T/C	0.62	0.270	22.0	78.00	Multiciliate cell development
	rs9290959	3	190,624,703	C/A	0.91	−0.366	13.3	70.67	
LSG1	rs4393849	3	194,478,411	A/G	0.68	0.213	10.3	71.33	Nuclear export GTPase
MSL2	rs1393786	3	135,854,035	C/A	0.74	0.236	14.1	70.67	DNA damage response
KCNK2	rs59084003	1	215,154,276	C/T	0.93	0.534	24.0	70.67	Two-pore-domain background potassium channel
	rs864736	1	215,150,260	C/A	0.45	0.310	30.3	68.00	
WNT3	rs199529	17	44,837,217	A/C	0.77	−0.165	8.1	66.00	Cell patterning in embryogenesis
KANSL1	rs2696671	17	44,316,888	A/G	0.78	−0.197	6.9	65.33	Histone acetylation
STAG1	rs13092193	3	136,240,366	T/C	0.76	0.208	10.5	62.67	Sister chromatid cohesion

In a similar direction, rs13089287, located 54 kb upstream of the geminin coiled-coil domain containing (*GMNC*) gene, is significantly associated with increased LBAG in the subcallosal area, orbitofrontal cortex, insula, and multiple subregions of the cingulate cortex (Fig. 5C). Likewise, rs1393786, located14 kb downstream of the MSL complex subunit 2 (*MSL2*) gene, exhibits significant widespread positive associations with LBAG, particularly in parietal, occipital, and sensorimotor regions, including the intraparietal sulcus, postcentral sulcus, angular gyrus, and central sulcus (Fig. 5D). These results indicate that, in addition to KCNK2, other variants in GMNC and MSL2 may act polygenically to promote an older-appearing cortical morphology. In contrast, rs12146713, located on intron 4 of the NUAK family kinase 1 (*NUAK1*) gene, exhibits significant widespread bilateral associations with LBAG, most prominently in the orbital sulci, pericallosal sulcus, insula, and medial and lateral temporal regions (Fig. 5E). This variant displays the broadest spatial distribution of significant associations among all SNPs examined, but with consistently negative β coefficients, suggesting that the reference allele is associated with a younger-appearing cortex. Overall, we found that *KCNK2* variants demonstrate the strongest positive associations with cortical aging, promoting an older-appearing cortex, potentially in concert with variants in *GMNC* and *MSL2*. Conversely, *NUAK1* exerts widespread opposing effects, highlighting that the polygenic architecture of cortical aging reflects a balance between pro-aging and geroprotective genetic influences.

### WNT- and neurotransmission-related variants act on cortical aging through regional effects

Several variants exhibited strong, region-specific associations without broad cortical effects. These SNPs (rs2707466, rs17279437, rs1880512, rs798489, and rs1409682) ranked among the top 10 highest-impact variants (Table 1) but did not rank among the top 10 broadest-impact variants (Table 2). Collectively, they exhibited positive, negative, or mixed *β* coefficients, suggesting that genetic influences may regulate cortical aging through regionally specific mechanisms.

**Fig. 6:**
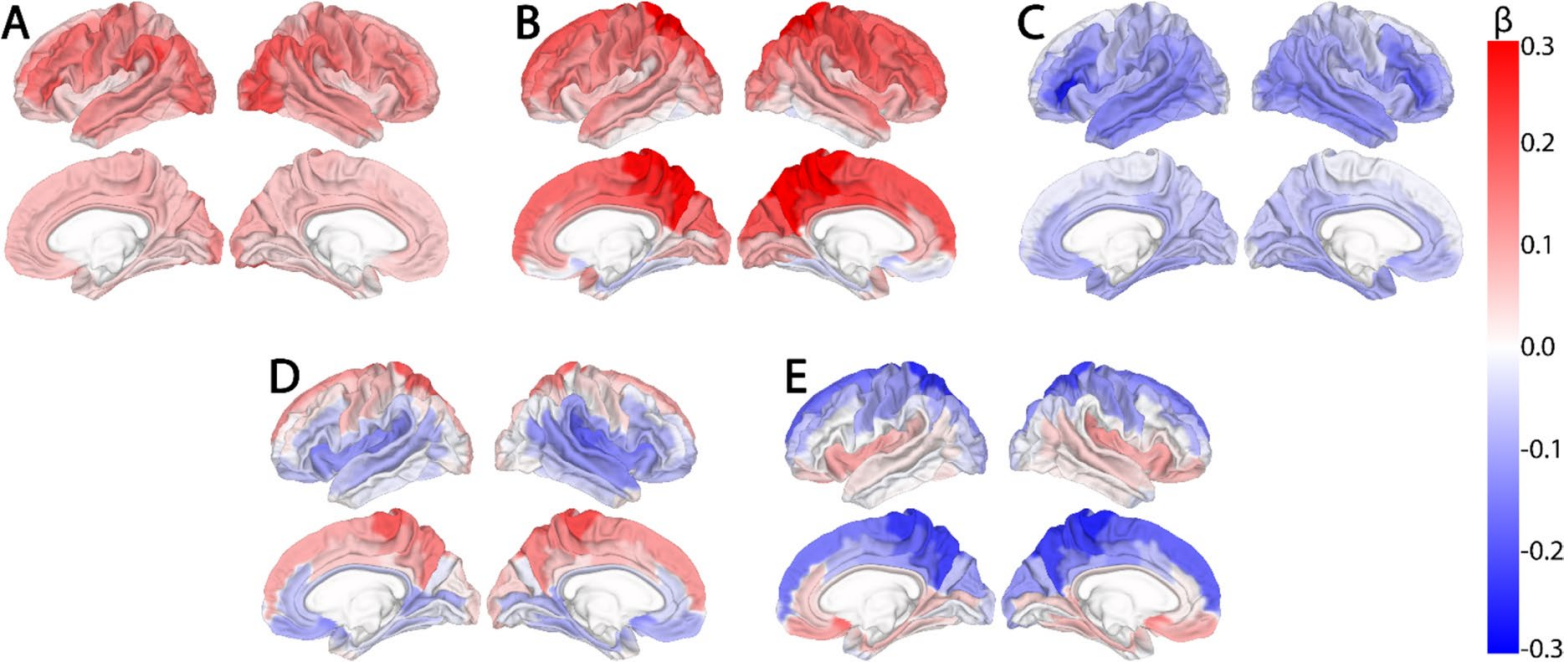
Cortical maps of β coefficients for highest-impact SNPs linked to LBAG. Red: positive associations (higher LBAG with more reference alleles); blue: anticorrelation. These SNPs include (**A**) rs2707466, WNT16; (**B**) rs17279437, SLC6A20; (**C**) rs1880512, SLC25A13; (**D**) rs798489, GNA12; and (**E**) rs1409682, LPAR1. All are ranked within the top 10 highest-impact SNPs but are not concurrently identified as broadest-impact variants

For example, rs2707466, located within exon 4 of the WNT family member 16 gene (*WNT16*), exhibits significant bilateral positive associations with LBAG in occipital, sensorimotor, and frontal regions (Fig. 6A). Similarly, rs17279437, within exon 5 of the solute carrier family 6 member 20 gene (*SLC6A20*), has significant positive associations across parietal, cingulate, and sensorimotor cortices (Fig. 6B). In contrast, rs1880512, located 26 kb upstream of the solute carrier family 25 member 13 gene (*SLC25A13*), exhibits significant negative associations in the orbitofrontal cortex, inferior and middle frontal gyri, lateral sulci, and temporal regions (Fig. 6C). Notably, rs798489, located within intron 2 of the G protein subunit alpha 12 gene (*GNA12*), displays a mixed pattern of association, positive in several parietal and frontal regions, including the paracentral lobule, precuneus, and superior frontal gyrus, but negative in temporal, insular, and subcallosal regions (Fig. 6D). Likewise, rs1409682, located within intron 5 of the lysophosphatidic acid receptor 1 gene (*LPAR1*), exhibits both positive and negative associations. However this SNP reached genome-wide significance only for decreased LBAG across bilateral parietal and frontal regions (Fig. 6E). Together, these findings indicate that variants within genes involved in WNT signaling (*WNT16*) and neurotransmission (*SLC6A20, LPAR1, SLC25A13, GNA12*) exhibit strong but spatially constrained associations with cortical aging. This suggests that the polygenic architecture of cortical aging reflects an interplay between broadly acting and regionally specialized genetic mechanisms.

### Cortical maintenance gene variants exhibit the broadest effects on brain aging

**Fig. 7:**
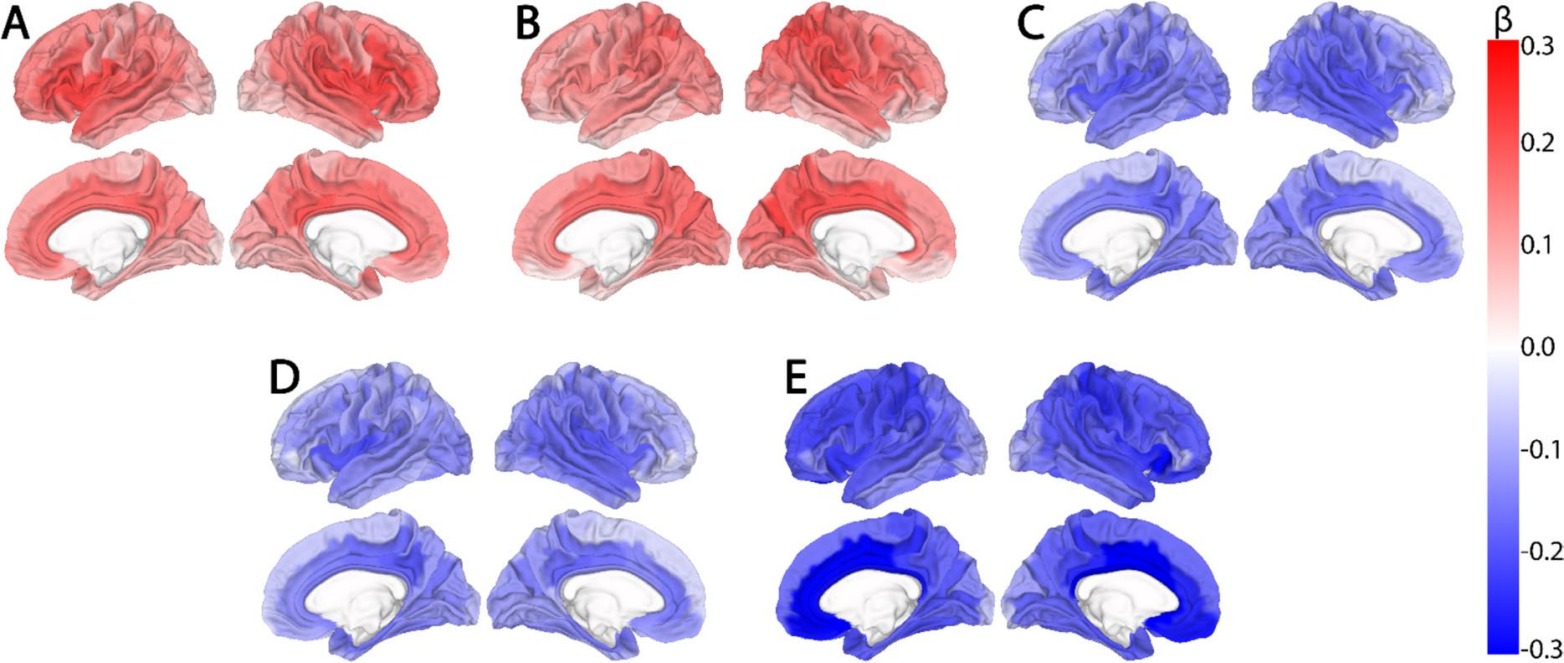
Cortical Maps of β Coefficients for broadest-impact SNPs linked to LBAG. Red: positive associations (higher LBAG with more reference alleles); blue: anticorrelation. These SNPs include (**A**) rs4393849, LSG1; (**B**) rs13092193, STAG1; (**C**) rs199529, WNT3; (**D**) rs2696671, KANSL1; and (**E**) rs9290959, GMNC. All are ranked within the top 10 broadest-impact SNPs but are not concurrently identified as highest-impact variants

In contrast to regionally constrained variants, several SNPs exhibited broad cortical associations without strong focal effects. These SNPs (rs4393849, rs13092193, rs199529, rs2696671, and rs9290959) ranked among the top 10 broadest-impact variants (Table 2) but not among the top 10 highest-impact variants (Table 1**)**. Like the highest-impact variants, they exhibited both positive and negative *β* coefficients, indicating bidirectional genetic influences on cortical aging. For example, rs4393849, located 31 kb upstream of the large 60S subunit nuclear export GTPase 1 (*LSG1*) gene, exhibits widespread significant positive associations with LBAG, most strongly in the insular, inferior frontal, and opercular cortices (Fig. 7A). Similarly, rs13092193, residing within intron 6 of the STAG1 cohesin complex component gene (*STAG1*), has broad bilateral positive associations with LBAG, notably in parietal and cingulate cortices (Fig. 7B). In contrast, rs199529, located 3 kb downstream of the WNT family member 3 (*WNT3*) gene, is marked by significant bilateral negative associations with LBAG, particularly in the central sulcus and adjacent sensorimotor regions (Fig. 7C). Likewise, rs2696671, located 43 kb upstream of the KAT8 regulatory NSL complex subunit 1 gene (*KANSL1*), exhibits widespread negative associations with LBAG in the insula, central sulcus, parietal lobes, and occipitotemporal regions (Fig. 7D). rs9290959, located 44 kb upstream of *GMNC*, also has significant widespread bilateral negative associations with LBAG, particularly in orbitofrontal and cingulate cortices (Fig. 7E).

Together, these findings suggest that genes involved in cortical maintenance—such as chromatin remodeling (*KANSL1*), cell-cycle regulation (*GMNC*), and protein synthesis and export (*LSG1, STAG1*)—contribute to broad, globally distributed effects on cortical aging. This contrasts with the spatially localized influences of WNT- and neurotransmission-related genes, highlighting that the polygenic architecture of cortical aging integrates both system-wide and region-specific genetic mechanisms.

### Dimensionality reduction reveals distinct cortical association patterns

**Fig. 8:**
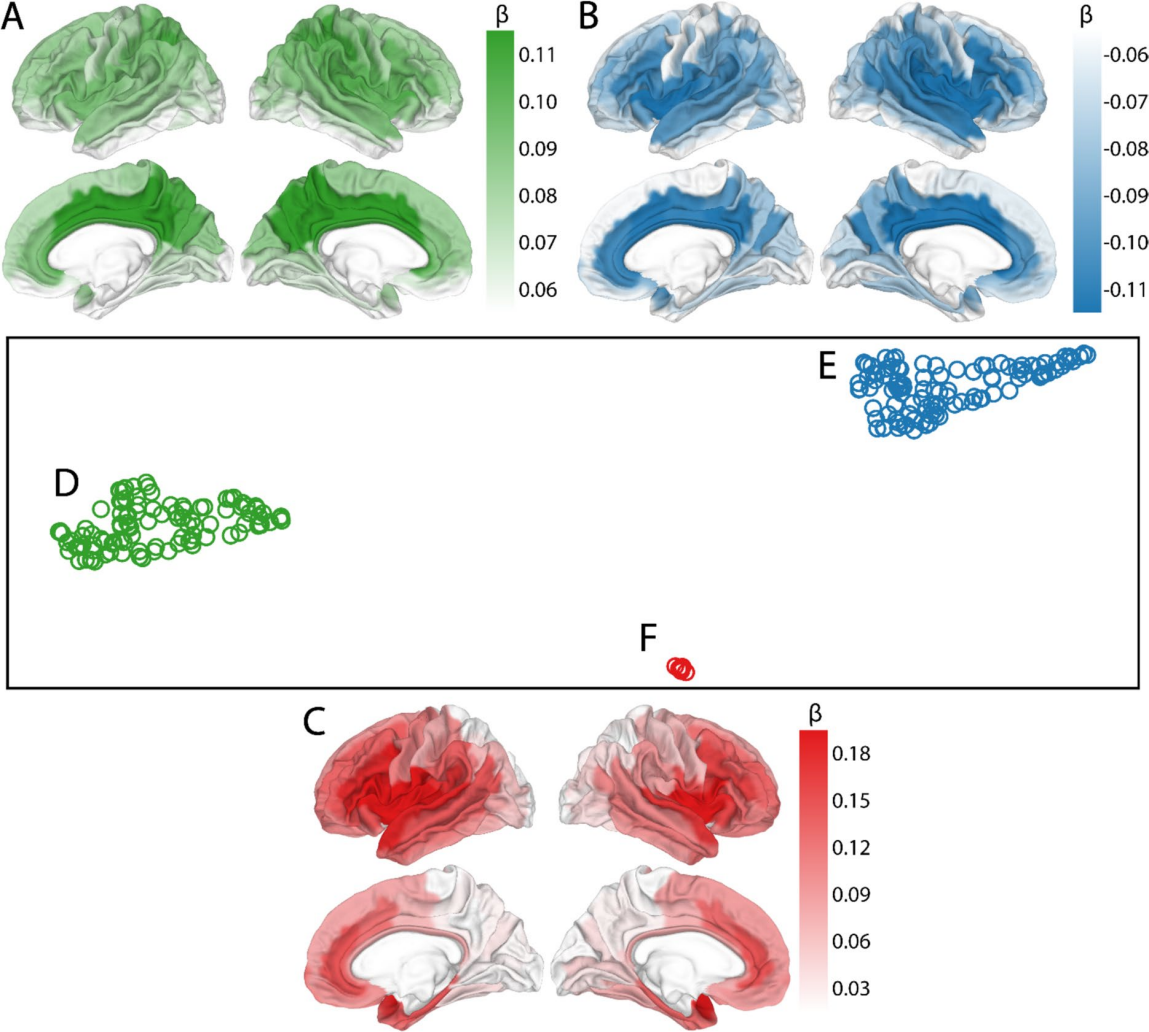
UMAP visualization of SNPs significantly associated with LBAG in more than seven cortical locations. Points’ proximity within clusters indicates the extent to which SNPs’ cortical patterns of association with LBAG are similar across the cortex. Each cortical overlay conveys the average association between LBAG and the SNPs in the respective cluster. The color scales indicate the averaged *β* coefficients for clustered SNPs, showing the direction and strength of association between their reference allele numbers and LBAG. Color coding (green. blue, and red) links each cluster in the UMAP to its respective cortical overlay. (A) represents the green SNP cluster (D); (B) the blue cluster (E); and (C) the red cluster (F)

To identify groups of SNPs with similar regional effects on LBAG, we applied uniform manifold approximation and projection (UMAP) followed by density-based spatial clustering of applications with noise (DBSCAN). UMAP is a nonlinear dimensionality reduction algorithm that projects high-dimensional data into a low-dimensional space while preserving both local and global structure, making it well suited for visualizing complex cortical association patterns. DBSCAN groups nearby points within this embedded space based on density, enabling detection of clusters with arbitrary shape without requiring the number of clusters to be predefined. Using this approach, we clustered the UMAP embedding of β coefficient cortical correlation maps for SNPs significantly associated with LBAG in more than seven cortical regions. This analysis resulted in three distinct groups (Fig. 8).

Group A (Fig. 8D) comprises 106 SNPs, including the key variants rs13089287, rs1393786, rs59084003, and rs864736, which rank among both the top 10 highest-impact and broadest-impact SNPs. This group also contains rs17279437 and rs2707466, which are exclusive to the highest-impact set, as well as rs13092193 and rs4393849, which are specific to the broadest-impact category. Genes most frequently mapped to variants in Group A—i.e., linked to the greatest number of SNPs in this cluster, as determined using the Stanford Genomic Regions Enrichment of Annotations Tool (GREAT) browser (see Methods)—are the *KCNK2*, PR/SET domain 6 (*PRDM6*), bone morphogenetic protein 6 (*BMP6*), ras homolog MTORC1 binding (*RHEB*), and protein kinase AMP-activated non-catalytic subunit gamma 2 (*PRKAG2*) genes, highlighting processes related to ion channel activity, vascular signaling, and cellular metabolism. When their cortical patterns are averaged across SNPs, the variants in Group A exhibit a widespread bilateral pattern of positive associations with LBAG, particularly concentrated in the midline and parietal structures. These cortical areas encompass the precuneus, posterior cingulate, and superior frontal regions, core components of the default mode and attention networks (Fig. 8A).

Group B (Fig. 8E) contains 106 SNPs, including the notable variant rs12146713, which ranks among both the highest-impact and broadest-impact SNPs. It also comprises rs1409682, rs1880512, and rs798489 (exclusive to the highest-impact set) and rs199529, rs2696671, and rs9290959 (unique to the broadest-impact category). Genes most frequently mapped to SNPs in Group B are the *NUAK1*, *LPAR1*, rho associated coiled-coil containing protein kinase 1 (*ROCK1*), A-kinase anchoring protein 13 (*AKAP13*), and insulin (*INS*) genes, implicate pathways related to cytoskeletal stability, lipid signaling, and stress response. When averaged across SNPs, the cortical association profile for Group B is marked by a widespread and bilateral pattern of negative *β* coefficients (Fig. 8B). The strongest inverse associations are concentrated in the limbic and paralimbic structures, including the insula, cingulate, and medial temporal regions—key hubs of emotional and interoceptive processing.

Group C (Fig. 8F) comprises only six SNPs and does not contain any variants ranked among the top 10 in either the highest-impact or broadest-impact categories. Genes most frequently mapped to Group C are the H1.1 linker histone, cluster member (*H1-1*), H4 clustered histone 8 (*H4C8*), tripartite motif containing 38 (*TRIM38*), zinc finger and SCAN domain containing 31 (*ZSCAN31*), butyrophilin subfamily 3 Member A2 (*BTN3A2*) genes, highlighting chromatin remodeling and immune-regulatory processes. When averaged across SNPs, the variants in Group C exhibit strong positive associations with LBAG in the frontoinsular and perisylvian regions, localized to the insular and perisylvian cortices that integrate sensory, cognitive, and affective information (Fig. 8C).

Together, these findings suggest that the polygenic architecture of cortical aging is organized along multiple, functionally distinct axes. A default mode-centered metabolic axis (Group A) promotes cortical aging through variants mapped to genes regulating ion channel activity, vascular signaling, and cellular metabolism. In contrast, a limbic-protective axis (Group B) supports cortical preservation through variants in genes involved in cytoskeletal stability, lipid signaling, and stress response. Finally, a frontoinsular integrative axis (Group C) mediates more localized aging effects, with variants mapped to genes implicated in chromatin remodeling and immune regulation. Collectively, this organization highlights that cortical aging reflects the coordinated influence of distinct, spatially and functionally specialized genetic systems distributed across large-scale brain networks.

## Discussion

### Significance

GBA is a widely studied biomarker of neurodegeneration, but previous genetic studies of GBA focus on a single global measure that fails to capture region-specific variation in cortical aging. Here, we present the first GWAS of LBA, using a novel deep learning model to estimate voxel-level LBA in up to 41,708 individuals. We identified 1,212 independent genetic variants associated with region-specific cortical aging, implicating genes involved in ion transport, cytoskeletal remodeling, mitochondrial metabolism, chromatin regulation, and neurodevelopmental signaling. Dimensionality reduction of *β* coefficient cortical correlation maps (via UMAP) followed by DBSCAN clustering identified groups of SNPs with similar patterns of cortical association with LBAG. These clusters are biologically coherent in that the genes mapped to SNPs within each group are enriched for morphogenetic, cytoskeletal, and immune-epigenetic processes, paralleling pathways previously implicated in AD, frontotemporal dementia (FTD), and related disorders. These findings define a polygenic framework for regional brain aging and support LBA as a genetically driven phenotype for studying the spatial heterogeneity of neurodegeneration. For example, SNP-informed polygenic scores for LBA could help researchers as they evaluate resilience vs. vulnerability to AD, or other diseases years before symptoms.

### Genetic architecture of LBA

LBA is not simply an anatomically descriptive measure of brain aging. Instead, it acts as a genetically informed *intermediate phenotype* between DNA and disease, offering finer resolution than traditional neuroimaging biomarkers. Specifically, our results reveal a series of coordinated genetic programs (developmental, metabolic, cytoskeletal, immuno-epigenetic) that choreograph brain aging over the life course. This supports the hypothesis that neurodegenerative disease risk is conditioned upon early development that later determines regional vulnerability to disease. Our research reconceptualizes brain aging as the unfolding of genetically encoded, partially-predictable trajectories, rather than as random atrophy or functional loss.

To reduce CA-related confounding and to isolate region-specific deviations from normative aging, we analyzed LBAG—the residual difference between predicted BA and CA—rather than raw LBA estimates. We identified 1,212 SNPs with significant genome-wide associations with LBAG in at least one cortical region. While a detailed discussion of all variants is beyond the scope of this study, many are mapped to genes involved in key biological processes such as transmembrane ion transport, chromatin regulation, neurodevelopmental signaling, and cytoskeletal dynamics. Notably, these pathways overlap with core mechanisms implicated in early neurodegenerative diseases [6–8].

### Genes involved in membrane transport and metabolic coupling broadly modulate cortical vulnerability to aging

Several top-ranking variants are mapped to genes involved in membrane transport, a pathway tightly coupled to neuronal excitability, osmotic balance, and mitochondrial function—all of which have been linked to changes in brain aging [9]. Disruptions in these processes are also key features of early neurodegenerative pathology, where ionic imbalance and metabolic stress contribute to synaptic dysfunction and neuronal loss [6]. Among our notable findings is the *KCNK2* gene, which is mapped to two independent genomic loci (rs864736 and rs59084003), both of which ranked within the top 10 for highest-impact and broadest-impact variants. We also found that rs864736 exhibits the most significant *β* coefficient for association with LBAG across all examined variants. Previous studies have associated both variants with multiple imaging-derived brain traits across the cortex, including *T*_*1*_-MRI gray-white matter contrast (GWC), cortical thickness, and cortical sulcal opening [10, 11]. These features are associated with cortical atrophy, a hallmark biomarker of neurodegenerative diseases such as AD [10, 11]. Additionally, a variant in linkage disequilibrium with rs864736 has been associated with alterations in GBA [12]. Together, these findings suggest that variation near KCNK2 may exert broad regulatory effects on cortical morphology and brain aging.

*KCNK2* encodes a two-pore domain potassium channel involved in passive transmembrane ion transport and has been found to regulate both neuronal excitability and inflammatory signaling [13, 14]. These functions are closely tied to brain aging: chronic neuronal hyperexcitability can promote excitotoxicity and metabolic stress, while persistent neuroinflammation can disrupt synaptic integrity and accelerate cortical atrophy [15, 16]. Notably, both *KCNK2* variants are associated with increased LBAG in cingulate, precuneus, and parietal regions—areas found to exhibit early vulnerability in AD [17, 18]. Reduced TREK-1 activity, encoded by *KCNK2*, has also been linked to increased neuronal excitability and calcium dysregulation, two key contributors to neurodegenerative progression [7, 19]. Widespread associations of variants mapped to *KCNK2* with increased LBAG may reflect its contribution to region-specific vulnerability through both electrical and immune-mediated pathways that drive structural brain aging and neurodegeneration.

Like *KCNK2*, the *SLC25A13* and *SLC6A20* genes also encode transport proteins. *SLC25A13* encodes a mitochondrial aspartate-glutamate carrier that facilitates calcium-sensitive glutamate transport, which supports the malate-aspartate shuttle and ATP production [20]. A variant mapped to this gene (rs1880512) was ranked among the highest-impact SNPs and associated with decreased LBAG across multiple frontal and temporal regions. Prior work has linked this variant to increased brain functional connectivity [21], suggesting that enhanced mitochondrial transport and metabolic coupling may contribute to preserved cortical function with age. Disruption of mitochondrial aspartate-glutamate exchange is also implicated in neuronal energy imbalance and oxidative stress—factors found to contribute to early neurodegenerative changes [20, 22, 23]. Furthermore, reduced interaction between Tau protein and *SLC25A13* has been observed in mutated Tau forms associated with neurodegenerative diseases, indicating potential mitochondrial dysfunction that could contribute to brain aging and neurodegeneration [23]. *SLC6A20* encodes a sodium- and chloride-dependent transporter for proline and glycine [24]. The variant rs17279437, mapped to this gene, was ranked among the highest-impact SNPs and was associated with increased LBAG. This association may reflect impaired synaptic stability or metabolic efficiency resulting from altered *SLC6A20*-mediated amino acid transport. Additionally, prior studies have found disruptions in proline and glycine homeostasis to impact neuronal signaling pathways and glial function [24], which may contribute to region-specific vulnerability observed in the aging cortex.

*LSG1*, also a transport-related gene, encodes a GTPase involved in the nuclear export of the 60S ribosomal subunit, which is essential for cytoplasmic protein synthesis [25]. The rs4393849 variant, mapped to *LSG1* and ranked among the broadest-impact SNPs, was associated with increased LBAG predominantly in the insular and frontal opercular regions. Deficits in ribosomal export can impair cellular protein production and stress resilience [26], two processes central to aging, and may thereby sensitize these regions to structural decline and neurodegeneration. Additionally, atrophy in the insular and opercular cortices has been linked to early neurodegenerative changes associated with impaired proteostasis and translational stress [8]. Furthermore, mitochondrial import and degradation of aggregation-prone proteins, including *LSG1*, is a critical process that maintains protein balance in cells and prevents mitochondrial dysfunction and cellular stress, which are key contributors to brain aging and neurodegeneration [27]. Notably, *LSG1* is highly expressed in microglia and astrocytes [28], suggesting that genetic variation at this locus may disrupt glial translational capacity and metabolic support of neurons. Because microglial activation and astrocytic dysfunction are key drivers of neuroinflammation and synaptic instability during aging, altered *LSG1*-mediated ribosomal export in these cell types may amplify regional vulnerability to neurodegenerative processes. However, these expression patterns do not exclude important effects in other neural cell populations, and it remains uncertain which cell types represent the primary mediators of *LSG1*-associated genetic risk, indicating the need for targeted follow-up studies using cell-type-specific eQTL analyses and spatial transcriptomics.

### Genes involved in chromatin remodeling and transcriptional control indicate an epigenetic axis of cortical aging

We found evidence linking genes involved in DNA regulation to LBAG. For instance, the *GMNC* gene is mapped to two variants—rs13089287, which was ranked within the top 10 for both highest-impact and broadest-impact variants, and rs9290959, which was ranked among the broadest-impact variants. Prior studies have associated rs13089287 with variation in GWC across the cortex, consistent with its widespread associations with increased LBAG observed in our study [10]. In contrast, rs9290959 was associated with decreased LBAG across cortical regions, suggesting opposing effects on regional brain aging. *GMNC* encodes a chromatin-binding protein involved in the regulation of DNA replication and cell cycle progression, particularly in progenitor cell populations [29]. Through this role in coordinating cellular proliferation and differentiation, *GMNC* may influence cortical development and tissue maintenance. These processes are known to shape cortical integrity and therefore may impact BA estimates derived from structural imaging [30]. The opposing effects of the two *GMNC*-linked variants also suggest allele-specific mechanisms may differentially modulate regional brain aging, either contributing to cortical preservation or increasing structural decline. Furthermore, variations in and near *GMNC* have been linked to increased AD risk and brain aging patterns characterized by alterations in fornix mean diffusivity on MRI [31, 32].

Like *GMNC*, the *MSL2* gene is also linked to chromatin regulation [33]. The rs1393786 variant is mapped to this gene and exhibits strong positive associations with LBAG as a highest-impact SNP. *MSL2* encodes a component of the male-specific lethal (MSL) complex, which plays a critical role in chromatin remodeling and histone acetylation—epigenetic mechanisms that regulate transcription and determine cell identity [33]. Unlike *GMNC*, which acts primarily during early cell cycle regulation, *MSL2* may influence cortical aging by modulating transcriptional landscapes in mature neural tissue. Dysregulated chromatin can disrupt the expression of genes critical for synaptic resilience, thereby promoting aging-related neurodegeneration [34]. Notably, rs1393786 has also been associated with metabolic traits such as cholesterol and triglyceride levels, indicating that lipid metabolism may intersect with epigenetic regulation in shaping cortical aging [35]. These findings support a model in which long-term cortical integrity is shaped by the interplay between metabolic and epigenetic pathways.

The *KANSL1* gene encodes a subunit of the NSL (nonspecific lethal) complex, which is also involved in histone acetylation and transcriptional regulation [36]. This gene is mapped to the rs2696671 variant, a broadest-impact SNP with widespread negative associations with LBAG. Like *MSL2*, *KANSL1* may exert its effects through chromatin remodeling and maintain cortical integrity through long-term epigenetic control. Variations in *KANSL1* have also been identified as risk loci for AD, PD, and ALS [37]. Similar to *KANSL1*, the *STAG1* gene encodes a component of the cohesin complex involved in sister chromatid cohesion and DNA repair [38]. The rs13092193 SNP is mapped to this gene and exhibited positive associations with LBAG as a broadest-impact variant, particularly in the parietal regions. Disruption of *STAG1* function has been implicated in genome instability and impaired neuronal maturation, both of which may increase cortical aging [34, 38]. Additionally, variation in *STAG1* has been implicated in a neurodevelopmental disorder characterized by cognitive impairment [39].

### Genes governing early cortical patterning continue to shape region-specific aging trajectories

Two members of the WNT signaling family—the *WNT3* and *WNT16* genes—were also implicated in our analysis. Both *WNT3* and *WNT16* encode a glycoprotein involved in canonical Wnt/*β*-catenin signaling, which is essential for embryonic patterning and neurogenesis [40]. *WNT3* is mapped to the rs199529 variant, which was ranked among the broadest-impact SNPs and is associated with decreased LBAG in the sensorimotor and temporal cortices. rs199529 has also been identified as a genetic risk locus for PD [41]. *WNT16* is mapped to the rs2707466 SNP, which exhibited widespread positive associations with LBAG as a highest-impact variant, particularly in the occipital and sensorimotor regions. rs2707466 has been linked to cortical volume changes in AD patients [42]. These results suggest that Wnt signaling may influence cortical aging in a regionally specific manner, potentially by regulating early developmental and neuroplasticity pathways.

### Genes involved in cytoskeletal organization, cell adhesion, and signaling regulate structural resilience of the aging cortex

Genes involved in cytoskeletal regulation emerge as potential contributors to variation in LBAG. The *NUAK1* gene encodes a serine/threonine kinase that regulates cytoskeletal remodeling, axonal integrity, and tau phosphorylation [43, 44]. The rs12146713 SNP is mapped to *NUAK1* and exhibited the most widespread associations with decreased LBAG across all examined variants. Prior studies have linked this variant to differences in GWC, medial thalamic nuclei volume, and sulcal widening, suggesting it may play a broader role in cortical development and morphogenesis [10, 45, 46]. rs12146713 has also been associated with changes in gray matter volume in brain regions particularly vulnerable to age-related degeneration and metabolic pathways implicated in neurodegeneration [47]. Additionally, age-related decline in cytoskeletal integrity has been linked to neurodegeneration and cognitive impairment [48]. *NUAK1* is known to regulate tau phosphorylation and microtubule stability, and its inhibition is currently under investigation as a therapeutic approach for tau-related disorders [49]. For example, a study in a fruit fly model demonstrated that suppressing *NUAK1* activity effectively mitigates neurodegeneration [44]. These findings indicate that variations in *NUAK1* may help preserve structural integrity and reduce LBAG by promoting cytoskeletal stability during neurodevelopment.

The *LPAR1* gene, which encodes a G-protein-coupled receptor activated by lysophosphatidic acid, has also been found to play a role cytoskeletal dynamics [50]. Prior research has implicated its encoded protein in modulating neurogenesis, white matter integrity, and actin filament organization [50]. The rs1409682 SNP, mapped to *LPAR1*, was identified as a highest-impact variant and associated with decreased LBAG in parietal and frontal cortices. Notably, this variant has also been linked to enhanced white matter connectivity [51]. These findings suggest that variations in *LPAR1* may contribute to a younger-appearing cortex by supporting myelination and neural plasticity through cytoskeletal regulation, akin to those in *NUAK1*. Furthermore, deletion of *LPAR1* has been associated with neurodevelopmental disorders and demyelinating diseases [52].

The *GNA12* gene encodes the alpha subunit of the G12 heterotrimeric G protein, which also regulates cytoskeletal organization, as well as cell adhesion and tight junction stability [53]. This gene is mapped to the rs798489 SNP, which was ranked among the highest-impact variants and exhibited mixed associations with LBAG. rs798489 was positively associated with LBAG in the parietal and frontal regions and negatively associated in the temporal and subcallosal cortices. Like *NUAK1* and *LPAR1*, variations in *GNA12* may influence cortical aging through cytoskeleton-mediated signaling pathways that govern cellular structure, adhesion, and neural plasticity. Variations in *GNA12* have also been linked to brain aging patterns involving changes in ventricular volume [32].

### Summary of GWAS findings

Our findings highlight several converging biological pathways associated with local brain aging. The most impactful variants were mapped to genes involved in ion and metabolite transport (e.g., *KCNK2*, *SLC25A13*, *SLC6A20*), chromatin regulation and DNA replication (e.g., *GMNC*, *MSL2*, *STAG1*, *KANSL1*), Wnt-mediated developmental signaling (e.g., *WNT3*, *WNT16*), and cytoskeletal regulation (e.g., *NUAK1*, *LPAR1*, *GNA12*). These pathways converge on mechanisms known to influence structural brain aging such as metabolic efficiency, neuroinflammation, chromatin accessibility, and cytoskeletal stability [15, 16, 34, 48]. Many of the implicated genes and pathways have also been linked to neurodegenerative diseases, including AD, PD, and ALS. Across these loci, we observed diverse associations with LBAG, ranging from widespread effects spanning multiple cortical networks to region-specific patterns confined to functionally specialized areas, reflecting heterogeneous genetic influences on the spatial distribution of brain aging. These findings support a model in which genetic variation contributes to individual differences in the pattern of brain aging, with certain alleles accelerating age-related decline across distributed networks while others confer resilience. Therefore, LBA may serve as a sensitive intermediate phenotype for detecting genetic influences that predispose cortical regions to early neurodegenerative change. Variants associated with elevated LBAG highlight candidate loci for accelerated aging and pathology, while those associated with decreased LBAG may reveal protective mechanisms preserving cortical integrity and delaying neurodegenerative processes.

### Dimensionality reduction identifies pathway-specific SNP clusters

UMAP and DBSCAN clustering of LBAG-linked SNPs identified three groups with distinct cortical association profiles, reflecting different modes of genetic influence on brain aging. These clusters exhibit both anatomical and functional coherence, indicating that variants with similar regional effects may act through shared biological pathways. This suggests that variation in cortical aging may result from coordinated genetic programs rather than random or isolated effects.

Group A SNPs—dominated by variants mapped to the *KCNK2*, *BMP6*, *PRDM6*, *RHEB*, and *PRKAG2* genes—exhibit a bilateral band of positive *β* coefficients centered on the medial parietal regions (precuneus, posterior cingulate, subparietal and marginal sulci). These are among the most metabolically active and structurally integrated regions of the cortex, making them particularly sensitive to subtle dysregulation over time [54–56]. The co-involvement of *KCNK2* (ion-channel regulation [13]), *BMP6* (bone morphogenetic signaling [57]), and *RHEB* (mTOR pathway [58]) suggests a shared theme of metabolic and morphogenetic control—indicating that genes governing early growth and long-term bioenergetics may be key modulators of aging in the aforementioned cortical hubs. The consistency of spatial patterns across SNPs in this group supports the idea that regional vulnerability in these areas may stem from a shared developmental or regulatory foundation.

Group B comprises protective or negative-LBAG SNPs mapped to genes such as *NUAK1*, *LPAR1*, *ROCK1*, *AKAP13*, and *INS*. These variants exhibit widespread negative *β* coefficients, especially in the anterior/middle cingulate, insula, and paralimbic cortices—regions involved in emotion regulation, interoception, and salience processing [59–61]. Genes mapped to these SNPs are involved in Rho-GTPase signaling, cytoskeletal remodeling, and membrane blebbing, reflecting a shared theme of structural resilience and intracellular signaling regulation. Notably, several genes in this group—such as *ROCK1* and *LPAR1*—directly modulate actin cytoskeleton dynamics and cellular tension, processes critical for synaptic maintenance and glial function [50, 62]. The co-occurrence of these pathways within a single SNP group suggests that maintaining cytoskeletal balance may be a protective strategy against cortical aging. This effect appears especially relevant in limbic and paralimbic networks prone to early degeneration in disorders like FTD.

Group C presents a distinct immuno-epigenetic theme. The six SNPs in this group map to genes involved in histone function and immune signaling (*H1-1*, *H4C8*, *TRIM38*, *ZSCAN31*, *BTN3A2*), and their cortical effect profiles are spatially restricted to the frontoinsular and perisylvian regions. These areas are critical for social, sensory, and affective integration and are highly interconnected, making them sensitive to inflammation and regulatory stress [61, 63]. Genes within this group are implicated in interferon-gamma signaling and viral entry regulation, suggesting that heightened immune activation or chromatin instability may contribute to accelerated aging in these cortical networks.

Altogether, this dimensionality reduction approach reveals that SNPs associated with LBAG may act in coordinated, biologically cohesive groups that selectively target specific cortical systems. These findings suggest that there may be at least three partially distinct genetic pathways of brain aging: (A) morphogenetic and metabolic regulation affecting default-mode network hubs, (B) cytoskeletal and signaling mechanisms supporting resilience in limbic and paralimbic structures, and (C) immune-epigenetic disruption localized to frontoinsular and salience regions. By identifying these clusters, we uncover higher-order patterns in how genetic variation contributes to region-specific vulnerability and highlight potential molecular entry points for targeted interventions.

### Limitations and future directions

This study presents the first GWAS of LBA, providing insight into the genetic underpinnings of region-specific brain aging. However, important avenues remain to be explored. First, while our sample size was large and well-powered for discovery, our cohort was limited primarily to individuals of European ancestry. Future studies should validate these findings in more heterogeneous, multi-ancestry populations to ensure the generalizability of LBAG-associated variants. Such research may uncover ancestry-specific loci that may contribute to different brain aging trajectories across populations.

Secondly, our cross-sectional design does not capture how LBA changes over time. Longitudinal imaging-genetics studies are needed to determine whether the identified SNPs and pathways estimate future changes in LBA or instead reflect stable anatomical traits. Tracking within-subject LBA trajectories could also clarify causal direction and whether certain variants actively drive aging in specific regions or early structural vulnerabilities shape genetic associations.

Thirdly, functional validation of top SNPs and gene clusters is necessary to move from association to mechanism. In vitro and in vivo modeling of high-impact variants—particularly those in *KCNK2*, *NUAK1*, *GMNC*, and *LPAR1*—could reveal how these genes alter neuronal excitability, chromatin state, or cytoskeletal dynamics to influence cortical aging. For instance, single-cell RNA-seq and epigenomic profiling in carriers of risk alleles may also help define cell-type specificity and timing of these effects.

Fourthly, expanding both the scope of genetic data and the depth of downstream molecular characterization could refine our understanding of the causal pathways linking genotype to LBA. Our analyses relied on array-based genotype data and did not incorporate whole-exome sequencing, whole-genome sequencing, or the full set of imputed variants (over 90 million) available in UKBB. While leveraging these resources could improve fine-mapping resolution and enable detection of rare coding or regulatory variants not well captured by genotyping arrays, such analyses would impose substantial computational demands. In particular, the need to estimate associations separately across 148 cortical regions would make genome-wide analyses of the full imputed dataset prohibitively intensive. Future work could address this challenge by focusing on region-prioritized subsets of variants, applying scalable statistical approaches, or leveraging cloud-based computational frameworks. Integrating high-resolution variant data with other omics layers—such as transcriptomics, methylomics, proteomics, and metabolomics—would further help map genetic associations to molecular intermediates. For example, linking LBAG-associated variants to expression quantitative trait loci (QTLs), protein QTLs, or methylation QTLs could reveal convergent biological pathways underlying cortical aging.

Finally, LBA as a neuroimaging phenotype holds translational potential. However, alternative interpretations of increased LBA have been noted in prior work [64]. For instance, elevated LBAG values may partly reflect statistical artifacts such as regression to the mean or residual dependence on chronological age, rather than true biological deviation. In such cases, observed group differences could arise from differences in age distributions or model calibration rather than reflecting accelerated cortical aging per se. In our present study, we mitigated this concern by including chronological age as a covariate, thereby regressing out linear age dependence from our association analyses.

Future work should test whether LBA, especially when indexed by SNP-informed polygenic scores, can predict cognitive decline, neurodegeneration, or response to treatment. Beyond risk prediction, our findings also raise the possibility that LBA could serve as a biomarker for patient stratification or treatment-response prediction in neurodegenerative disease trials. Several of the pathways implicated by our GWAS—including Wnt signaling, cytoskeletal regulation, chromatin remodeling, and mTOR-related metabolic control—are already targets of active or emerging therapeutic strategies [65–68]. Because LBA provides a regionally resolved index of cortical aging, it may capture treatment-sensitive biological variation that is obscured by global brain age measures. For example, individuals with genetically elevated LBAG in default mode or limbic networks may represent subgroups with heightened vulnerability to Alzheimer’s disease-related neurodegeneration, whereas those with protective genetic profiles in cytoskeletal or lipid signaling pathways may exhibit greater treatment resilience. In this context, LBA could be leveraged as a stratification tool to enrich clinical trials for individuals most likely to show progression, or as a quantitative imaging endpoint to measure regional treatment effects over time. Longitudinal and interventional studies will be needed to determine the sensitivity of LBA to disease-modifying therapies and to support its utility as a pharmacodynamic biomarker.

An additional important direction for future work will be to resolve the cell-type-specific mechanisms underlying LBAG-associated variants. For instance, although *LSG1* exhibits enriched expression in microglia and astrocytes [28], the present study cannot determine whether these glial populations represent the primary cellular mediators of genetic risk, nor can it exclude important effects in neuronal or vascular-associated cell types. Integrating LBA-associated variants with cell-type-resolved eQTL datasets and single-cell or single-nucleus transcriptomic atlases from human cortex will be critical for clarifying these relationships. Such integrative analyses could determine whether ribosomal export-related genetic effects act through excitatory neurons, inhibitory interneurons, glial populations, or neuroimmune pathways, and help explain why these genes exhibit regionally patterned associations with cortical aging. Future studies combining spatial transcriptomics with imaging-genetics will be particularly valuable for linking genetic risk to regional and cell-type-specific molecular processes that drive vulnerability to neurodegeneration.

Beyond its use as a stratification or monitoring biomarker in clinical trials targeting age-related cortical decline, additional study designs could further refine its clinical utility. For instance, predictive modeling studies could evaluate whether incorporating LBA alongside established imaging, cognitive, and fluid biomarkers improves individualized risk prediction for neurodegenerative outcomes. Large-scale biobank analyses integrating electronic health records may also clarify how LBA relates to broader health trajectories and comorbidities in aging populations. Finally, network neuroscience approaches—such as connectome-wide association studies or structural–functional coupling analyses—could assess how LBA patterns align with large-scale brain network organization, offering insights into systems-level vulnerability and resilience in cortical aging. Together, these directions could advance both the mechanistic understanding and clinical application of LBA as a tool for studying brain aging and neurodegeneration.

## Conclusions

This study presents the first genome-wide investigation of LBA, a spatially resolved biomarker of cortical aging that captures regional variation overlooked by global BA metrics. Through the integration of deep learning-based neuroimaging, GWAS, and unsupervised clustering, we establish a framework for linking common genetic variation to the anatomical heterogeneity of brain aging. Our findings reveal that regional brain aging is governed by a polygenic architecture enriched for genes involved in ion transport, cytoskeletal remodeling, mitochondrial metabolism, chromatin regulation, and neurodevelopmental signaling. These pathways converge on cellular processes implicated in neurodegeneration. Dimensionality reduction clustered SNPs into biologically coherent groups, such as morphogenesis, metabolic regulation, cytoskeletal stability, and immune-epigenetic control. This suggests that cortical aging is not driven by isolated variants, but by coordinated genetic programs active from development through late life. Furthermore, LBA may provide a biological bridge between normative aging variability and neuropathological decline. Future work integrating LBA with longitudinal, multiomic, and network neuroscience data will be key to translating these findings into clinical applications. By uncovering the genetic logic of cortical aging, this study contributes to a more precise understanding of how the brain changes over time and how those changes might be accelerated or delayed.

## Materials and methods

### Participants

This study analyzed data from the UKBB, a large-scale biomedical database (http://www.ukbiobank.ac.uk), under application number 47656. The study cohort included 41,859 cognitively normal individuals (21,983 females) aged 45 to 83 years (mean: 64.4 years; standard deviation: 7.7 years), with a sex ratio of 1.11 females per male. Further details on UKBB infrastructure and participant recruitment can be found in [69].

### MRI acquisition and preprocessing

3D volumetric *T*_*1*_*-*weighted MRIs were acquired using a standard Siemens Skyra 3.0 T scanner with a voxel size of 1 mm × 1 mm × 1 mm and a matrix size of 208 × 256 × 256 voxels. FreeSurfer’s recon-all function was used to reconstruct and segment MRIs. This process included skull-stripping, motion correction, normalization of nonuniform signal intensities, Talairach space transformation, removal of non-brain tissues, and registration of subjects' brains into the MNI305 atlas. FreeSurfer (FS) was selected for several reasons: (1) FS reconstructions are readily available from UKBB, (2) its fully automated workflow enhances efficiency, and (3) it supports surface analyses and registrations across both native and atlas spaces.

### DNN for LBA estimation

**Fig. 9:**
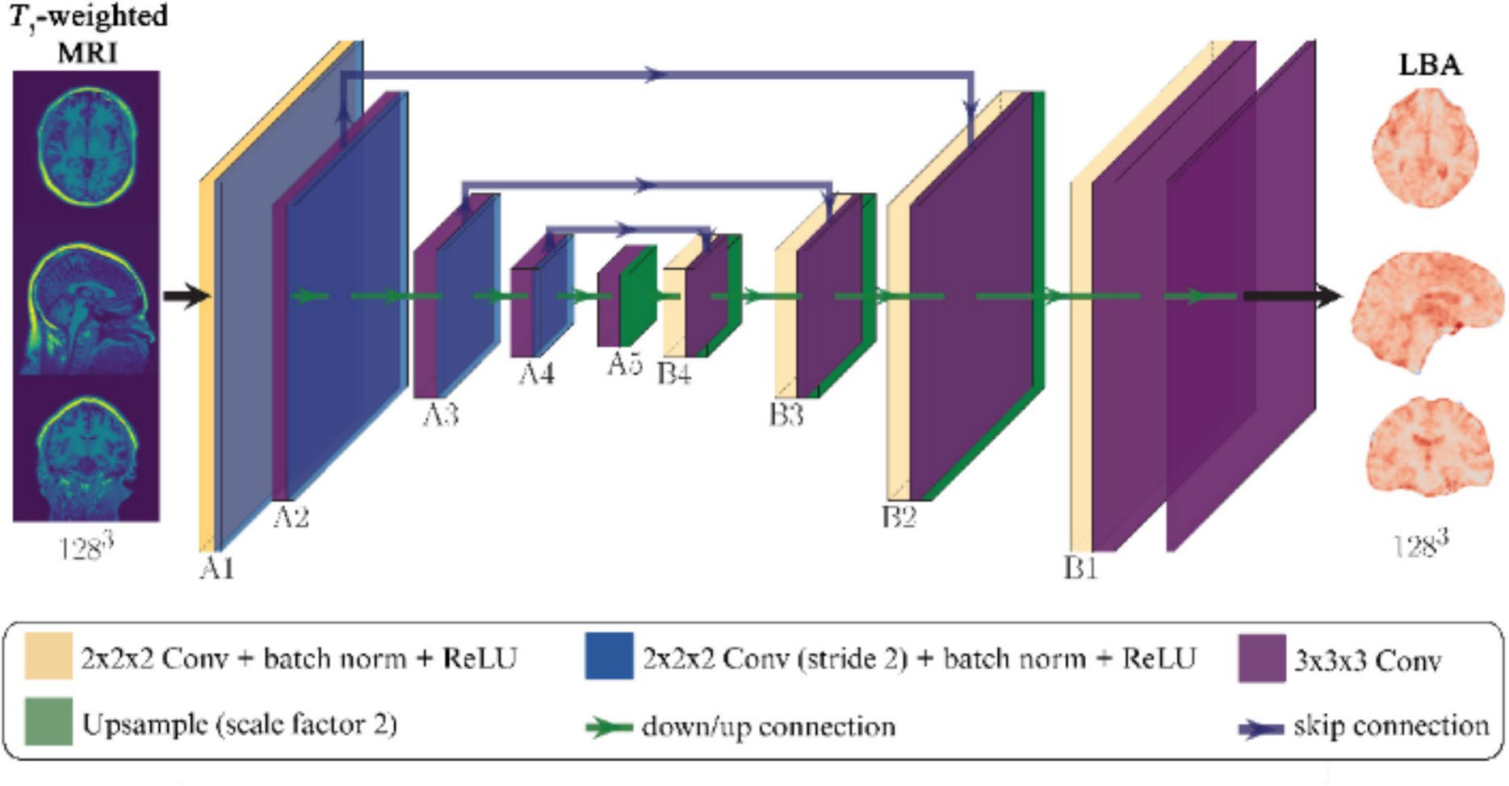
Overview of the AE-CNN for voxel-wise LBA prediction from *T*_*1*_-weighted MRI. The model comprises an encoder (A1–A5), bottleneck (latent feature space), and decoder (B4–B1) built on a 3D CNN backbone. The encoder progressively compresses the input volume using strided convolutional blocks (Conv) with increasing filter depth (16 to 256), composed of 3D convolutional layers with batch normalization and ReLU activation. A1 contains initial 2 × 2 × 2 convolutional layers, while A2–A4 include stacked 3 × 3 × 3 convolutional layers followed by 2 × 2 × 2 strided convolutions. The resulting latent representation of size 8^3^ is decoded through a symmetric expansion path using nearest-neighbor up sampling and 3 × 3 × 3 conv layers, with decreasing filter depth (128 to 16). Skip connections link A4 to B4, A3 to B3, and A2 to B2, preserving spatial detail across scales. A final convolutional layer generates the predicted single-channel BA volume of size 128^3^. The model is trained using a voxel-wise MAE loss between predicted BA and ground-truth CA

Our team independently designed, implemented, trained, and validated an auto-encoder 3D convolutional neural network: a deep learning regression model designed to estimate LBA at the voxel level. Given a 3D preprocessed MRI scan *x* and a corresponding 3D ground-truth *y* representing CA, the model estimates a voxel-wise LBA volume *y’*. All three volumes (input MRI, ground-truth CA, and estimated LBA) are of the same size, with the ground truth assigned as the subject’s CA at each brain voxel or set to 0 otherwise. The DNN comprises an encoder and a decoder built with 3D-convolutional neural networks. The overall framework is illustrated in Fig. 9.

*T*_*1*_-weighted, skull-stripped brain MRIs (volume size 128^3^) are processed through five convolutional blocks (A1-A5). A1 consists of a 3D convolutional layer with kernel size 2, stride 1, and padding to preserve spatial dimensions, followed by batch normalization and ReLU activation. This is followed by a 3D convolution with kernel size 2 and stride 2 to downsample the volume by half, again followed by batch normalization and ReLU. Subsequent encoder blocks (A2-A4) each contain three 3D convolutional layers (kernel size 3, stride 1), a downsampling convolution (kernel size 2, stride 2), batch normalization, and ReLU activation. The number of filters in each block increases with depth: 16 in A1, 32 in A2, 64 in A3, 128 in A4, and 256 in A5, enabling hierarchical feature extraction. After A5, the input volume is compressed into a latent representation of size 8^3^.

The decoder reconstructs the BA map from this compressed latent representation using four blocks (B4-B1). Each decoder block includes nearest-neighbor upsampling to double the volume dimensions, followed by 3D convolutions (kernel size 3, stride 1), batch normalization, and ReLU activation. Nearest-neighbor interpolation is chosen over transposed convolutions to avoid checkerboard artifacts in the output. The number of filters decreases symmetrically: 128 in B4, 64 in B3, 32 in B2, and 16 in B1. Skip connections link encoder and decoder blocks with matching spatial dimensions to preserve fine structural information: specifically, A4 is connected to B4, A3 to B3, and A2 to B2. A final convolutional layer collapses the feature maps across channels to output a volume of size 128^3^ containing single-channel, voxel-wise LBA estimates.

The model is trained using a mean absolute error (MAE) loss function computed only over voxels inside the brain. Optimization is performed using the Adam optimizer with a learning rate of 0.0005. The model is trained for up to 500 epochs with a batch size of 64, with early stopping applied if validation performance does not improve over 10 consecutive epochs. ReLU activation is applied to all convolutional and dense layers to ensure non-linearity and reduce vanishing gradients. Batch normalization regularizes training and accelerates convergence. The model is implemented in Python 3.7 using PyTorch 1.13. All experiments are conducted on a workstation equipped with a 128-core CPU, 512 GB RAM, and an A100-80 GB GPU.

Regression to the mean can introduce a CA-dependent bias in estimated LBAs, similar to effects observed in previous studies [70, 71]. To mitigate this bias, we applied the zero-correlation constraint method [70] to adjust LBA estimations for participants in the test set. To quantify region-specific deviations in LBA, we computed LBAG by subtracting each participant’s CA from their bias-corrected LBA estimates. Higher LBAG values indicate that a brain region appears older than expected for a given CA, serving as a regional marker of accelerated brain aging. Alternative interpretations of elevated LBAG are also possible, which are acknowledged in the Discussion under Limitations. LBAG values were mapped onto the FS Destrieux cortical atlas to account for inter-individual variability in cortical morphology and parcellated into 148 anatomically defined regions (e.g., right precentral gyrus) using FS automated labeling as described in [72]. All subsequent analyses were performed using these LBAG values.

### Genetic data

Genetic data for UKBB participants were generated using array-based genotyping. A total of 488,377 participants were genotyped using two custom Affymetrix arrays: the UK BiLEVE Axiom array (49,950 participants) and the UK Biobank Axiom array (438,427 participants), which together directly assayed approximately 800,000 markers across the genome [73] (UKBB Category 263). For the present study, we limited analyses to 784,036 directly genotyped SNPs to balance genome-wide coverage with computational feasibility, as GWAS was conducted separately for each of 148 cortical regions. Quality control excluded (A) 18,876 SNPs available in fewer than 35,000 participants, (B) 95,267 SNPs where over 99% of participants were monomorphic, (C) 4,873 SNPs with missing reference identifiers, and (D) 2,049 SNPs with incorrect reference allele information. The final analysis included 662,971 SNPs.

Participant genotypes were coded based on the number of reference alleles (0, 1, or 2) present at each SNP. SNPs were mapped to genes using the GREAT browser [74], implemented via a Selenium-based workflow (https://github.com/SamAndTheSun/greatbrowser) [75]. GREAT associates genomic regions with putative target genes using a regulatory domain model that assigns each gene a basal regulatory domain (5 kb upstream and 1 kb downstream of the transcription start site), which is then extended up to 1 Mb in both directions until it reaches the basal domain of a neighboring gene. SNPs were mapped to the nearest gene within this extended domain, with ties resolved in favor of downstream genes. This approach enables functional annotation of both coding SNPs (directly mapped to overlapping genes) and noncoding SNPs (linked to nearby genes that they may regulate via distal interactions).

### GWAS

Linear regression was used to evaluate the dose–response relationship between (A) the number of reference alleles at a given SNP and (B) LBAG. Covariates included age at MRI scan (UKBB fields 34, 52, and 53), sex (field 31), assessment center (field 54), and the first five genetic principal components to account for population structure (field 22009) [76]. Associations were calculated for all 148 cortical regions. Since genotype data were not available for all 662,971 SNPs in every participant, sample sizes were adjusted per SNP, excluding individuals with missing data. Consequently, the sample sizes for GWAS ranged from 34,922 to 41,708 participants (mean sample size: 41,360; standard deviation: 812). Under the null hypothesis of no linear correlation, *p*-values were computed using a two-tailed t-test comparing *β* regression coefficients.

SNPs were clumped using PLINK software (https://www.cog-genomics.org/plink/1.9) [77] to identify independent association signals. Clumping is an LD-based method that selects representative SNPs by grouping highly correlated SNPs within a specified genomic window. The process involves defining an index SNP based on its significance level and then removing SNPs in high LD within a set genomic distance. This approach ensures that highly correlated SNPs do not inflate association signals while preserving the most informative genetic variants. We defined index SNPs as those with *p* < 0.05 and removed SNPs in high LD (*r*^2^ > 0.1) within a 250 kb window. Clumping was performed separately for each chromosome, reducing the dataset to 379,470 SNPs. For each cortical region, the genomic inflation factor (λ) was calculated from the median chi-square statistic of all SNPs to assess test statistic inflation. Genome-wide significance was evaluated using *p* < 0.05 with Benjamini–Hochberg correction to control FDR. All subsequent analyses used this clumped, FDR-corrected SNP set.

Phenograms illustrating significant genetic associations for each cortical region relative to SNP genomic locations were generated using the Ritchie Lab’s online visualization tool (https://visualization.ritchielab.org/pheno-grams/plot). A connectogram displaying pairwise connections between cortical regions that shared at least 30 SNPs significantly associated with LBAG was generated using custom Python scripts and visualized with Circos software. Additional details on connectogram construction and interpretation can be found in [78].

SNPs were ranked based on (A) their most significant *β* coefficient and (B) their number of significantly associated cortical regions. A Manhattan plot visualized the *p-*values of these correlations and their SNP genomic positions. Rankings were based on Benjamini–Hochberg corrected *p-*values. For clarity, "highest-impact SNPs" refers to the 10 SNPs with the most significant *β* coefficients, while "broadest-impact SNPs" refers to the 10 SNPs with the largest number of significantly associated cortical locations. Coverage was defined as the percentage of the cortex where associations were statistically significant. Cortical maps of *β* coefficients were generated for SNPs from each ranking.

### Dimensionality reduction

UMAP is a dimensionality reduction technique that preserves the global and local structure of high-dimensional data, making it useful for clustering and visualizing complex patterns [79]. It operates by constructing a high-dimensional graph representation of the data and then optimizing a lower-dimensional layout that maintains the original data relationships. Unlike principal component analysis, which assumes linear relationships, UMAP captures nonlinear patterns, making it particularly effective for genomic and neuroimaging data.

*β* coefficient cortical correlation maps of SNPs that exhibited significant associations with LBAG in more than 7 cortical regions (220 SNPs) were analyzed using UMAP. UMAP was performed using the following parameters: 15 neighbors, 2 components, a minimum distance of 0.1, and a Euclidean metric. To group SNPs with similar cortical association patterns, we applied DBSCAN to the 2D UMAP-embedded data [80]. To estimate an appropriate epsilon (*ε*) parameter for DBSCAN, we first computed the distance to each point’s 5th-nearest neighbor— a common practice in DBSCAN applications for 2D data that balances sensitivity to small, dense clusters while reducing the impact of noise. The sorted *k*-distance values were then plotted to identify the inflection point in the *k*-distance graph, providing a heuristic for selecting an appropriate *ε* threshold. DBSCAN was then applied to form clusters based on this threshold. This method identified clusters of SNPs with similar cortical association patterns with LBAG. To determine the characteristic association pattern for each cluster, the *β* coefficients for each cortical region were averaged across SNPs within each group and visualized as cortical maps for each cluster. Genes mapped to SNPs within each cluster were then examined in the literature to identify common biological pathways and processes.

## Supplementary Information

Below is the link to the electronic supplementary material.

Supplementary file1 (CSV 163 KB)

Supplementary file2 (DOCX 27 KB)

## Data Availability

Qualified researchers may access the de-identified imaging, genetic, and demographic data used in this study through the UK Biobank (UKBB; http://www.ukbiobank.ac.uk), subject to an approved application and compliance with UKBB data access policies. MRI data were preprocessed, and LBAG values were mapped onto an average cortical atlas using FreeSurfer (https://surfer.nmr.mgh.harvard.edu). Linkage disequilibrium (LD) analysis was performed using PLINK (https://www.cog-genomics.org/plink/1.9). Post-GWAS analyses incorporated resources from the Stanford GREAT Browser (https://great.stanford.edu/great/public/html/) and UMAP for dimensionality reduction (https://github.com/lmcinnes/umap). All GWAS results are provided within the manuscript and accompanying supplementary materials.
