# Supplementary material for: Deep neural networks and genome-wide associations reveal the polygenic architecture of local brain aging
Source: GeroScience. 2025 Dec 11;48(3):3729–53. doi: 10.1007/s11357-025-02046-1 (PMC13356123; doi:10.1007/s11357-025-02046-1)
Supplement: Supplementary file 2 — Supplementary file2 (DOCX 27 KB) [file 11357_2025_2046_MOESM2_ESM.docx]

**Supplemental Information for**

**Deep neural networks and genome-wide associations reveal the polygenic architecture of local brain aging**


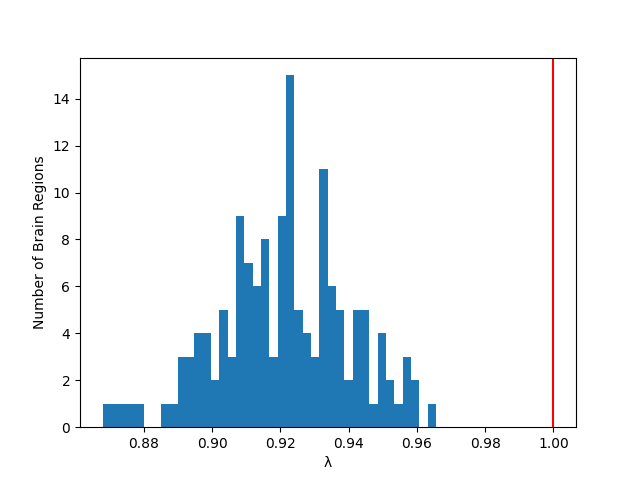


**Supplementary Fig. 1** Histogram of genomic inflation factors (λ) across cortical regions. The mean λ was 0.92 (range: 0.87–0.97), indicating slight deflation of test statistics. The x-axis represents λ values and the y-axis represents the number of cortical regions.
